# Hematopoietic Stem and Progenitor Cells (HSPCs) and Hematopoietic Microenvironment: Molecular and Bioinformatic Studies of the Zebrafish Models

**DOI:** 10.3390/ijms23137285

**Published:** 2022-06-30

**Authors:** Muhammad Faisal, Mubashir Hassan, Aman Kumar, Muhammad Zubair, Muhammad Jamal, Harish Menghwar, Muhammad Saad, Andrzej Kloczkowski

**Affiliations:** 1 Division of Hematology, College of Medicine, The Ohio State University Comprehensive Cancer Center, Columbus, OH 43210, USA; muhammad.faisal@osumc.edu; 2 The Steve and Cindy Rasmussen Institute for Genomic Medicine, The Research Institute at Nationwide Children’s Hospital, Columbus, OH 43205, USA; mubasher.hassan@nationwidechildrens.org; 3 Department of Ophthalmology and Vision Sciences, The Ohio State University, Columbus, OH 43210, USA; aman.jain1424@gmail.com; 4 Department of Veterinary Medicine, Jiangsu Academy of Agricultural Sciences, Nanjing 210014, China; zubair_durani@webmail.hzau.edu.cn; 5 Department of Immunology, School of Basic Medical Science, Wuhan University, Wuhan 430072, China; jamalbiotech@yahoo.com; 6 Axe Molecular Endocrinology and Nephrology, CHU de Quebec-Research Center (CHUL), Laval University, Quebec City, QC G1V 4G2, Canada; ham776@usask.ca; 7 Department of Animal Sciences, The Ohio State University, Columbus, OH 43205, USA; msaad@cuvas.edu.pk; 8 Department of Pediatrics, The Ohio State University, Columbus, OH 43205, USA

**Keywords:** hematopoietic stem cells (HSCs), hematopoietic niche, hematopoietic microenvironment, zebrafish, RNA-binding proteins

## Abstract

Hematopoietic stem cells (HSCs) reside in a specialized microenvironment in a peculiar anatomic location which regulates the maintenance of stem cells and controls its functions. Recent scientific progress in experimental technologies have enabled the specific detection of epigenetic factors responsible for the maintenance and quiescence of the hematopoietic niche, which has improved our knowledge of regulatory mechanisms. The aberrant role of RNA-binding proteins and their impact on the disruption of stem cell biology have been reported by a number of recent studies. Despite recent modernization in hematopoietic microenvironment research avenues, our comprehension of the signaling mechanisms and interactive pathways responsible for integration of the hematopoietic niche is still limited. In the past few decades, zebrafish usage with regards to exploratory studies of the hematopoietic niche has expanded our knowledge for deeper understanding of novel cellular interactions. This review provides an update on the functional roles of different genetic and epigenetic factors and molecular signaling events at different sections of the hematopoietic microenvironment. The explorations of different molecular approaches and interventions of latest web-based tools being used are also outlined. This will help us to get more mechanistic insights and develop therapeutic options for the malignancies.

## 1. Introduction

**Figure 1:**
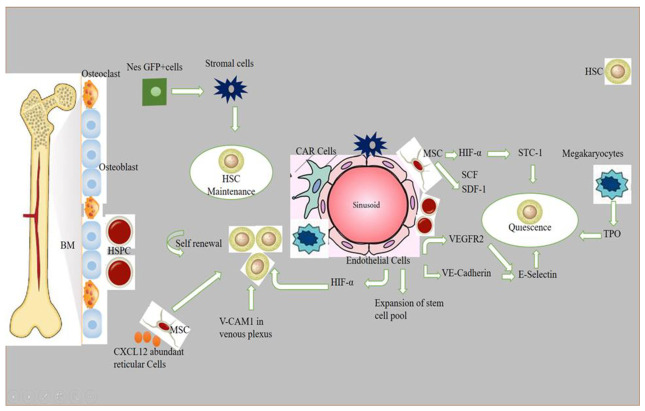
Role of different signaling factors responsible for hematopoietic niche maintenance and quiescence. A brief description of how normal HSPCs compete for microenvironmental space and resources. Niche cells, cytokines, signals, ECM, and oxygen gradient govern HSC activity. HSCs vary by subniche. Endosteal niches maintain LT-HSCs, while sinusoidal niches aid in hematopoietic development and regeneration. NG2+ arte-riolar pericytes block HSCs from shutting arterioles. LepR-expressing perisinusoidal cells produce SCF and CXCL12, required for HSC maintenance or mobilization. Different niches and subniches serve hematopoiesis.

Stem cell niches comprise a specialized microenvironment that promotes stem cell maintenance and regulates its function. HSC niches are perivascular in the spleen and bone marrow, and certain endothelial cells and stromal cells secrete factors which promote the maintenance and regulation of HSC niches [1]. Recent progress in the field helped to identify the cellular composition of hematopoietic stem and progenitor cells (HSPC), exploring the complex molecular networks that regulate the HSPC [2]. HSCs produce a variety of hematopoietic lineage cells in a specific microenvironment in bone marrow (BM) called “niche”. Multiple cells in BM contribute to HSC niche activity, and, among these, stromal cells are closely associated with vasculature [3]. The contribution of osteoblasts in HSC maintenance is still debatable, although the role of bone-derived molecules (e.g., osteopontin) or the role of bone turnover on HSC localization and function was demonstrated [4,5]. Studies have reported that the deletion of niche factors (CXCL12) or stem cell factors (SCF) from mature osteoblasts and osteoblastic progenitor cells does not lead to a reduction of HSCs in bone marrow (BM) [6,7,8]. Chimeric antigen receptor (CAR) cells express a high amount of CXCL12 and SCF, and are mainly distributed around sinusoids, and in the form of a homogenous tangled network in BM. CAR cell depletion using CXCL12 diphtheria toin receptor (DTR) results in a reduction of HSCs in BM [9]. Conditional deletion of CXCL12 from lepR-Cre marked cells mobilizes HSCs from BM to the spleen and peripheral blood, and LepR+ stromal cells around sinusoids have been shown to regulate the mobilization of HSCs pool [6]. Nes-GFP+ cells have also been identified as niche player in BM. Stromal cells within the population of Nes-GFP+ are an important source of niche factors critical for the maintenance of HSC [3,10]. Perivascular cells also express increased levels of major niche factors associated with HSCs [10,11]. When HSPCs arrive in a perivascular niche, then a group of endothelial cells (ECs) remodel and surround a single HSPC attached to a single mesenchymal stromal cell. These mesenchymal stromal cells anchor HSPCs and orient their divisions. A compound called lycorine promotes HSPC and niche interaction during development, which expands the pool of stem cells into adulthood [12]. ECs are part of the niche components. The blockade of angiogenic activity of ECs by neutralizing vascular endothelial cadherin (VE-cadherin) and vascular endothelial growth factor receptor-2 (VEGFR-2) impairs supportive function of ECs to HSCs [13]. HSC quiescence is also regulated by ECs via surface molecule E-selectin expression [14]. Conditional deletion of CXCL12 and SCF from ECs can decrease number of HSC in BM, and suggests a role of ECs in the maintenance of HSCs by producing these niche factors [6]. However, the heterogeneity of EC populations is unresolved. ECs with an increased expression of CD31 (CD31hi) and endomucin (Emcnhi), referred to as type H endothelium, are found in end-terminal arterioles connecting to sinusoids that express Kitl encoding SCF at higher levels than sinusoidal type L ECs [15]. However, the specific contribution of EC subset still requires further analyses with selective genetic deletion of SCF. Vascular permeability difference shows a differential role of ECs between sinusoids and arterioles influencing HSC niche. Due to the reduced permeability of arterial vessels which keep HSCs in low reactive oxygen species (ROS), HSCs are manifested in a quiescent state. On the contrary, more leaky sinusoids expose HSCs to blood plasma and promote a high level of ROS in HSCs, increasing the ability of differentiation and migration [16]. The whole mechanism of hematopoietic maintenance and quiescence, and factors governing its regulation at different anatomic locations of hematopoietic niches, are shown in Figure 1.

Non-myelinated Schwann cells wrapping the sympathetic nerves maintain HSC quiescence by activating TGFβ. Sympathetic signals induced by granulocyte colony-forming factor (G-CSF) also play a role in HSC mobilization from niche cells [17]. Macrophages are also considered as an important element of niche-modulating cells in BM, and the deletion of macrophages have shown HSPC mobilization into blood with a reduction of niche factor encoding genes [18]. Macrophages in BM participate in the regulation of HSC through the BM microenvironment [3]. The vascular cell adhesion molecule-1 (VCAM-1) macrophage-like niche cell population in the inner surface of venous plexus interacts with HSPCs in an integrin subunit alpha 4 (ITGA4)-dependent manner, and has its role in HSPC retention within the microenvironment [19]. Megakaryocytes (MKs), if selectively depleted, lead to a loss of quiescence of HSCs, and injection of cxcl4 produced by MKs increases quiescence, which leads to HSC reduction [20]. The removal of MKs results in an increased number of HSC, and proliferation and reduction of TGF-β1 protein and nuclear-localized phosphorylated SMAD2/3 in HSCs [21]. MKs regulate HSC quiescence by producing thrombopoietin (TPO), which is a crucial cytokine for HSC quiescence, and is mediated by membrane protein C-type lectin-like receptor-2 (CLEC-2) signaling [22,23]. The use of zebrafish to study the hematopoietic niche has enabled discoveries of novel cell-to-cell interactions and important regulators of HSCs, and the mystery of niche components may contribute to therapeutic efforts to direct differentiation of HSCs to improve stem cell transplants and to sustain stem cells in culture [24].

This review compiles the role of different genetic and epigenetic signaling interventions which are responsible for hematopoietic niche maintenance and quiescence. Moreover, we also present the advances of the latest technologies in hematopoietic microenvironment research avenues, and the latest web-tools using CRISPR/cas9 technology to explore it. Finally, we also present future perspectives and challenges related to research on HSCs niche.

A brief description of how normal HSPCs compete for microenvironmental space and resources: niche cells, cytokines, signals, ECM, and oxygen gradient govern HSC activity. HSCs vary by subniche. Endosteal niches maintain LT-HSCs, whereas sinusoidal niches aid in hematopoietic development and regeneration. NG2+ arteriolar pericytes block HSCs from shutting arterioles. LepR-expressing perisinusoidal cells produce SCF and CXCL12, required for HSC maintenance or mobilization. Different niches and subniches serve hematopoiesis.

## 2. Role of Epigenetic Factors Responsible for Hematopoietic Niche in Zebrafish Models

The zebrafish model is widely used to study the hematopoietic system, and has helped in the identification of various hematopoietic regulators. Recent studies on epigenetic regulation have enabled researchers to understand normal and malignant hematopoiesis [25]. Gene expression is controlled by chromatin conformation, and, if deregulated, then malignancies may occur [26]. The zebrafish serves as an excellent model to explore the mechanisms underlying chromatin regulation, and to evaluate the effects of chromatin-modifying drugs. Moreover, chromatin immunoprecipitation (chip) can be used in combination with sequencing to identify gene regulatory elements, chromatin architecture, and DNA binding sites in zebrafish [27]. Nuclear architecture protein cohesion and CCCTC binding factor (CTCF) contribute to gene regulation and chromatin structure. Cohesion is important for zygotic genome activation (ZGA). It is suggested that a subunit of cohesis Rad21, if depleted, causes a delay in ZGA; on the contrary, the depletion of CTCF affects little. Rad21 depletion destroys nucleoli formation and RNA polymerase II foci, leading to defective chromosome architecture [28]. Single cell RNA sequencing (ScRNA-seq), combined with ATAC-seq and immunophenotypic analysis, helps to integrate lineage differentiation with regulatory element accessibility [29].

Recent studies have shown that epigenetic modifications maintain hematopoietic cell fate by DNA methylation [30]. Dynamic changes in DNA methylation have been observed during cellular differentiation and development. Tissue-specific, differentially methylated regions (DMRs) overlap tissue-specific regulatory elements. The methylation pattern of developmental-stage-specific DMRs revealed a much stronger correlation than promoter methylation [31]. Hence, the developmental enhancer and DNA methylation exhibit an important status during zebrafish early development. However, the direct significance of the DNA methylation state of enhancers is unclear for most of the loci [32].

Studies have shown that epigenetic and epitranscriptomic factors are vital for reshaping gene expression patterns of hemogenic endothelial cells, as they are involved in HSC production [33]. Endothelial-to-hematopoietic transition (EHT) is required to generate HSCs, and it is brought about by transcription factors and signaling pathways, whereas Gata2 and Notch transcription factors are upstream regulators which are functionally followed by cMyb and Runx1 transcription factors [34]. These EHT genes represent DNA and RNA methylation, histone modifications, and chromatin remodeling as epigenetic mechanisms controlling the production of HSCs [33]. Moreover, epigenetic modification enables the maintenance of stem cell differentiation and development [35]. DNA methylation is an epigenetic mark, and has its role in the development of HSCs by the induction of transcriptional silencing [36,37]. Ge et al. have shown that HSC formation from the endothelium by EHT requires the ten-eleven translocation (Tet) family of cytosine dehydrogenases (tet1, tet2, Tet3), out of which, only Tet2 and Tet3 localize to the aorta gonad mesonephros (AGM) region, where HSCs bud off into the circulatory system [38]. DNA methyltransferase 1 (Dnmt1) is important in regulating gene expression by maintaining DNA methylation patterns, and it also maintains the HSPCs population in zebrafish [39].

Moreover, post-translational histone modifications in nucleosome give additional means in regulating chromatin accessibility, and it is a common mechanism to manage endothelial cells’ identity in HSC development [33,35]. Commonly, histone modifications include methylation and acetylation of lysine (K) residues on the N-terminal of the histone tail, and acetylation is carried out by histone acetyl transferases (HATs), which leads to the opening up of transcriptionally active chromatin states. On the contrary, deacetylation is carried out by histone deacetylases (HDACs), and it is responsible for gene inactivation through chromatin compaction. However, histone methylation has more effects on gene regulation that usually depend on the position of the lysine residue to be methylated [36]. Polycomb repressive complex (PRC) 1 is the earliest known epigenetic regulator of HSC formation, and works as an inhibitor for hemogenic EC specification [40]. In the later stages of HSC development, an epigenetic machinery CoREST repressive complex regulates EC identity [41].

Another type of epigenetic mechanism involved in HSC development regulation is chromatin remodeling. This process involves multi-subunit complexes which recognize the genomic landscape by ATP utilization, change the nucleosome position by sliding, and use eviction to change the nucleosome composition, followed by the reassembly of histone variants, and the resulting nucleosome compaction or expansion restricts or promotes the accessibility of transcription factors to regulatory regions [42]. Chromodomain helicase DNA-binding (CHD) is chromatin remodeling ATPase, and regulates HSC formation by the maturation of developing HSCs by increasing transcriptional output at the pro-hematopoietic gene level [33]. Overall, the role of epigenetic factors in hematopoietic niche maintenance plays a pivotal role, but further exploration is needed on the transcriptomic level and at post-transitional level to delineate the underlying mechanisms.

## 3. Effects of Different Signaling Events in Zebrafish Models: Activation or Repression

### 3.1. Signaling Events Getting into Niche Cells (BMP, Notch1, WNT)

Many important pathways, such as BMP, Notch, WNT, and sonic hedgehog (Shh) signaling, have been well documented in the context of the regulation of HSC by the mesenchymal stem cell (MSC); however, the modulatory events are yet to be resolved [43]. The regulation of hematopoiesis is performed by HSC in coordination with the secretion of multiple soluble factors which are involved in chemo-attraction, migration, signaling induction, proliferation, and the maintenance of HSC, and the interaction of signaling networks between HSC and MSC is vital in maintaining niche homeostasis. Recently, it has been shown that BMP4 can act on HSC directly or through downstream mediators, such as Shh, which induces HSC proliferation [44]. BMP affects HSCS development in embryos. Subtypes of HSC are thought to be controlled differentially by BMP signaling pathway. If BMPR1α is conditionally inactivated, it tends to increase the number of bone marrow (BM) HSCs and their homing and engraftment by inhibition of SMAD-dependent BMP signaling [45]. It has been shown that the BM microenvironment promotes the pro-lymphoid gene program in the HSCs subset activated by the BMP signaling pathway; moreover, the BMP signaling axis is the basis for HSCs heterogeneity [46]. Furthermore, BMP are considered critically important during bone formation, and are also involved in hematopoiesis, angiogenesis, and organogenesis during development. Moreover, deregulation of the BMP pathway may lead to hematological malignancies [47,48]. Multiple cells within the BM niche, including stromal cells, megakaryocytes, platelets, osteoblasts, HSCs, and hematopoietic cells, produce BMPs. Among BMPs, BMP2, BMP4, BMP6, and BMP7 act on MSCs, whereas stromal cell sand osteoblast precursor cells induce differentiation [49,50]. Precursors of BMPs direct protein to the secretory pathway, and dimerize due to post-translational modification, and then generate active homo- or heterodimers, followed by proteolytic cleavage. BMPs, once secreted, bind to specific receptors (type 1 and type 2) and activate R-SMADs, which make a complex with SMAD4, and translocate to the nucleus to initiate the transcription of target genes through binding with BMP responsive elements (BRE) in the promoter region [51].

Notch signaling has an important role in hematopoiesis and endothelial development, and Notch1 signaling is sufficient for the development of the hematopoietic niche. Notch signaling is considered important for HSC renewal, and the development of the BM endothelium [52]. Niche cells activate Notch signaling in HSPCs to enhance self-renewal and regenerative capacity [53]. Similarly, BM epithelial cells express Notch signaling which is regulated by proinflammatory stimuli [54]. The bone marrow endothelial cell line (BMEC) expresses jagged ligands, which expand hematopoietic progenitors and HSPCs expansion. Proinflammatory stimuli are also considered to increase expression of jagged 2 on Notch 1 and Notch 2 receptors [13]. Four Notch receptors (Notch 1–4) and five ligands (jagged 1 and 2; and Delta-like 1, 3, and 4) have been identified so far. These are single-span transmembrane proteins, and require cell-to-cell contact for activation. Soon after a Notch ligand is attached to Notch, cleavage is performed by the tumor necrosis factor and activated Notch intracellular domain (NICD), which is released followed by a second intercellular cleavage that initiates the signal. A molecular complex with secretase activity cleaves NICD, which helps in its translocation to the nucleus [55,56].

The regulation of stem cells, including HSCs in stem cell niches, is also performed by WNT signaling. The WNT ligand, when it binds to the Frizzled (Fz) receptor at the cell surface, inhibits phosphorylation and degradation of beta-catenin. This stabilized β-catenin then translocates to the nucleus, and binds to transcription factors to activate target genes [57]. Cross-talk between WNT and Notch signaling stabilizes beta-catenin on stromal cells, and promotes the self-renewal and maintenance of HSC in niches [58]. The balance between proliferation and quiescence is maintained to regulate the cell cycle fate of HSC. Combinations of receptors and ligands activate WNT signaling between HSC and MSC by canonical or non-canonical pathways; however, the role of WNT in relation to HSC is debatable [59]. It is released from MSC and exerts a paracrine effect on HSC quiescence, which is mediated by p21 up-regulation. WNT 3a expression in HSC down-regulates kit ligand, CXCL12, VCAM1, and angiopoietin-1 [60]. These data encompass the role of different signaling events getting into niche cells, but further investigations regarding modulation events and interactive pathway strategies among these signaling events need to be elucidated.

### 3.2. Signaling Factors Intermediating through Blood Cells

Chemokines were first described as being responsible for dictating leukocytes migration and activation; however, a chemokine ligand 12 (CXCL12), also known as stromal cell-derived factor 1, and its receptor, CXCR4, are among the first chemokines and receptors which are critical for the developmental process and homing and maintaining HSCs [61]. HSCs are mobile and generate blood cells. Firstly, they are generated from hemogenic endothelial cells of dorsal aorta in the mid-gestation period, and move to the fetal liver, and later, these HSCc and hematopoietic progenitors migrate and colonize bone marrow. It has been reported that CXCL12-CXCR4 signaling is important for bone marrow colonization by HSPCs [62]. CXCL12 abundant reticular (CAR) cells and endothelial cells are vital components of the hematopoietic niche; furthermore, nestin negative CAR cells, leptin receptor positive cells (LepR+), nestin green fluorescent protein positive mesenchymal progenitors, and PαS cells may contribute to the maintenance of HSC and B cell development [6,8]. CAR cells and LepR+ cells are important components of the HSCs niche and lymphoid progenitors, and they express specific transcriptors, which include Ebf3, Foxc1, and cytokines, including stem cell factor (SCF) and CXCL12, vital for niche functions. Moreover, macrophages and megakaryocytes are also involved in the maintenance of HSC [63]. The microenvironment in BM nurtures the pool of HSCs, and this quiescent HSC pool is maintained by CXCL12-CXCR4 signaling [64]. Chemokine family members are an important component of the cytokine network in BM, and control the retention, proliferation, and mobilization of hematopoietic progenitors [65].

Hemogenic epithelial cells are generated from dorsal aorta, and then, definitive HSCs enter the blood circulation and populate an intermediate hematopoietic niche before colonizing BM. In zebrafish, this niche is the caudal hematopoietic tissue. Followed by expansion, HSCs finally colonize in the kidneys [66,67,68].

### 3.3. Cellular Intrinsic Signaling Factors Responsible for Hematopoiesis

Multiple cell types, secretory factors, and adhesion molecules are associated with the HSC niche and directly affect stem cell behavior. Gap junctions in the bone marrow lymphoblastic hematopoietic niche control signaling functions, affect intracellular mechanisms, and improve cellular bioenergetics, and are involved in the regeneration of hematopoiesis [69]. P21 and EGr3 are intrinsic factors involved in blocking the differentiation of normal HSCs in acute myeloid leukemia due to the binding of SMAD3, an active transducer of transforming growth factor β1 (TGFβ1), to Egr3 [70]. Epoxyeicosatrienoic (EETs) and prostaglandin E2 (PGE2) are inflammatory molecules which are activated endogenously during hematopoietic regeneration; these lipid mediators are produced from niche cells. An interaction between HSCs and niche cells forms localized pockets that may increase the concentration of local growth factors and signaling molecules effectively. PGE2, EET, and other signaling pathways, including BMP, WNT, and Notch, tend to control HSC differentiation, self-renewal, and regeneration. Small lipid ligands and ligands from signaling pathways are up-regulated in stem cell niches during the regeneration of the hematopoietic system [71,72]. Elevated levels of cell-specific regulators (RUNX1, SCL, MYB, and LMO2) have been reported due to stimulation of the BMP and WNT pathways, suggesting role of these pathways in the intrinsic hematopoietic program during regeneration [73]. Signaling molecules, including BMP, WNT, EET, and PGE2, are involved during self-renewal and regeneration of HSC [74]. These secretory factors increase regeneration by the activation of transcription factors. However, determining all cellular factors in the niche and evaluating their behavior is important to understand the behavior of regenerative stem cells [73].

### 3.4. Molecular Signaling and Therapeutic Implications in Hematopoietic Malignancies

Acute myeloid leukemia (AML) disturbs the normal process of the production of blood cells, and renders patients with anemia, hemorrhage, and infections; as a result, the differentiation and proliferation of HSPCs are impeded in AML-infiltrated BM. Transcriptionally remodeled MSCs produce some secretory factors which suppress HSPC. Functional validation and secretome analysis studies showed that MSC-derived stanniocalcin (STC1) and transcriptional factor HIF-1α are limiting factors for the proliferation of HSPCs [75]. Changes in protein–protein and DNA–protein interactions and abnormal chromatin remodeling are at the root of uncontrolled gene transcription activation of signaling pathways in tumor cells [76]. Thienotriazolodiazepine has shown to induce apoptosis in non-GCB (germinal center of B cell) subtypes of diffuse large B cell lymphoma (DLBCL) by altering MYC- and E2F1-dependent gene expression by down-regulating the expression of signaling proteins of family toll-like receptor (TLR), NF-κB, and Janus kinase/signal transducers and activators of transcription (JAK/STAT) pathways, including TLR6, IRAK1, IRF4, MYD88, TNFRSF17, and IL6 [77]. An important regulator of AML cell autophagy is bromodomain containing protein 4 (BRD4), which works either by direct modulation of autophagy-related genes, or by increasing ROS species release by KEAP1 (kelch-like ECH-associated protein 1), followed by blocking the NRF2 (nuclear factor, erythroid 2-like 2) antioxidant pathway [78]. The recently discovered BETi, named INCB054329, has anti-proliferative effects over 32 cell lines of hematological malignancies, which include NHLs, AML, and MMs, and also showed a reduction of tumor growth for OPM2 myeloma cell lines. This effect was shown due to BRD4 displacement from FGFR3, MYC, NSD2, and IL6R enhancers, and the increased sensitivity of myeloma cells to JAK inhibitors [79]. Janus kinase 2 (JAK2) kinase inhibitors drive the development of myeloproliferative neoplasms (MPN). Members of the PIM family of serine/threonine kinases promote cellular proliferation and regulate apoptosis. Their overexpression is oncogenic, and they have been shown to induce lymphomas in collaboration with c-Myc; hence, PIM kinases are potential targets for solid tumors and blood cancers [80].

In the case of acute promyelotic leukemia (APL), SUMOYlation and destruction of the PML-RARα fusion oncoprotein is triggered by arsenic trioxide and used as front-line treatment in combination with retinoic acid. Arsenic induces SUMO-induced degradation [81]. Oncogenic RAS mutations occur in various leukemia; however, this effect is obtained by direct transformation via constant RAS/MEK/ERK signaling or an inflammation-related effect of KRAS. There exists a link between KRASG12D and NLRP3 inflammasome activation; studies have reported myeloproliferation and cytopenia in the active expression of KrasG12D. Therapeutic IL-1 receptor blockade and NLRP3 inhibition has shown reduced myeloproliferation and improved hematopoiesis, which is due to the activation of NLRP3 inflammosome due to ROS production in response to KrasG12D-RAC1 activation. Oncogenic KRAS not only acts by its canonical oncogenic driver function, but also enhances activation of the pro-inflammatory RAC1/ROS/NLRP3/IL-1β axis. This serves as a therapeutic approach based on immune modulation via NLRP3 blockade in KRAS-mutant myeloid malignancies [82]. Clonal expansion of T-cell and B-cell precursor leads to acute lymphoblastic leukemia, which is a hematologic neoplastic disorder. WNT/β-catenin pathway is a signaling axis involved in some physiological processes, for example, differentiation, development, and adult tissue homeostasis. Resultantly, deregulation of this signaling network is involved in the transformation of healthy HSCs in leukemic stem cells (LSCs), as well as cancer cell multi-drug-resistance [83]. TRAF-interacting protein with a forkhead-associated domain B (TIFAB) is implicated in myeloid malignancies, and its expression in hematopoietic stem/progenitor cells (HSPCs) allows USP15 signaling to substrates (MDM2 and KEAP1), and alleviates p53 expression. As such, TIFAB-deficient HSPCs show compromised USP15 signaling, and are sensitized to hematopoietic stress by repressing p53. The deletion of TIFAB in MLL-AF9 leukemia increases p53 signaling and consistently decreases leukemic cell function, leading to leukemia. Restoring USP15 expression somewhat rescues the function of TIFAB-deficient MLL-AF9 cells. On the contrary, elevated TIFAB represses p53 and increases leukemic progenitor function, and correlates with MLL gene expression programs in leukemia patients [84]. These findings highlight the recent role of molecular signaling events, and the roles of a variety of genes playing their part in therapeutic implications.

## 4. Role of RNA-Binding Proteins in Hematopoietic Niche

Hematopoiesis is a process by which mature blood cells are generated to carry out vital functions of the body. This process is maintained by a population of cells capable of self-renewal, hematopoietic stem cells, sustained in the bone marrow microenvironment, and having vascular endothelial cells and leptin receptor-positive (LepR+) mesenchymal stromal cells [44,85]. In zebrafish, HSCs emerge from the dorsal aorta, and, after reaching the caudal hematopoietic tissue (CHT), interact with caudal epithelial cells (cECs). Altogether, HSC niche in zebrafish is a complex cellular network involving epithelial cells, as well as stromal cells [12]. This interaction tends to drive a contact between HSCs and stromal cells to induce the proliferation of HSCs [12,86]. These stromal cells are known to express CXCL12a, which has a role in the retention of HSCs [8]. Another protein, Ebf2, has its role in establishing the osteoblastic niche in bone marrow, and controls the expression of genes involved in the maintenance of HSCs by contributing to the HSC niche [87]. Furthermore, the expression of CXCL12a is controlled by Foxc1 in stromal cells [8,88]. Moreover, ATF4 also plays a similar role in stromal cells [89]. Tfec belongs to the mitf family, and is a basic helix loop helix transcription factor. It is expressed in posterior blood island by the cECs forming the CHT niche, and is involved in myeloid biology [90,91,92]. Recent studies have reported the role of Tfec in HSC development and expansion at the non-cell autonomous level by modulating kitlgb expression in the vascular niche. Tfec-P2, which is produced from a distinct transcriptional starter site and is abundantly expressed in the CECs of zebrafish embryos, controls the hematopoietic niche [93].

**Figure 2:**
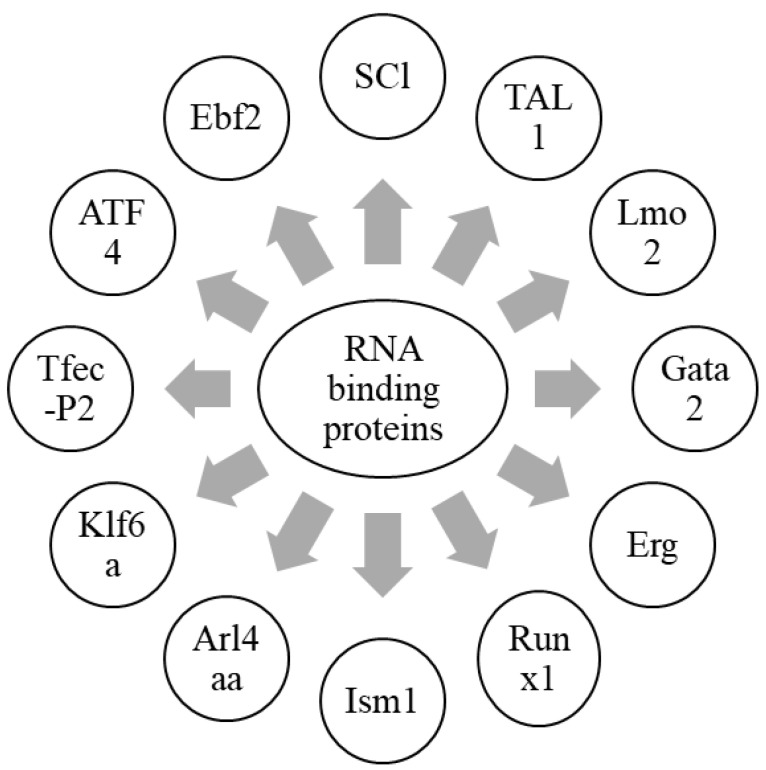
Graphical representation of RNA-binding proteins in hematopoietic niche.

Sinusoidal epithelial cells, mesenchymal stem cells, and perivascular cells are essential cellular components of the HSC niche. The kidney is a major hematopoietic organ in zebrafish, and is enriched with a double positive fraction of gata2a, which includes GFP and runx1: mCherry (gata2a + runx1+). These cells are associated with kidney sinusoidal epithelium. Gata2a-runx1+ cells abundantly contain erythroid- and/or myeloid-primed progenitors [94]. Krüppel-like factor 6a (Klf6a) is an EC-expressed transcription factor, and is essential for the CHT niche. It directly regulates the expression of chemokine (C-C motif) ligand 25b by modulating hematopoietic stem and progenitor cells (HSPCs) lodgment and proliferation, and thus, maintains hematopoiesis and vasculogenesis [95]. Furthermore, the knockdown of klf6a in zebrafish leads to the blockage of the maturation of primitive erythropoiesis [96]. ADP-ribosylation factor-like 4aa (Arl4aa), a member of the ADP-ribosylation factor family, is expressed in the ventral wall of the dorsal aorta and hematopoietic tissue during embryonic development. This protein is responsible for the initiation of definitive HSCs by maintaining Golgi complex integrity and Notch signaling [97]. The transcription factor, Runx1, expressed in the hemogenic epithelium (HE), is vital for the initiation of HSCs and endothelial-to-hematopoietic transition (EHT) in zebrafish by repressing the endothelial program through up-regulation of transcriptional repressors, Gfi1/Gfi1b [98,99]. Isthmin 1 (ism1) is essential for the normal generation of HSPCs in zebrafish hematopoiesis, and its knockdown leads to reduced numbers of erythrocytes, neutrophils, and macrophages [100]. SCl, TAL1, Lmo2, Gata2, Erg, and Runx1are responsible for the fate conversion of fibroblasts to hematoendothelial cells in hematopoietic programming [101]. These findings highlight different RNA-binding proteins which are involved in the HSC niche (Figure 2). In conclusion, RNA-binding proteins are important to initiate HSCs and maintain hematopoiesis, in collaboration with a variety of HSC niche factors.

Different RNA-binding proteins which are involved in the formation of HSCs, along with the maintenance of hematopoiesis, are summarized in this figure. Together with a wide array of HSC niche factors, RNA-binding proteins are an essential component in the formation of HSCs, as well as the maintenance of hematopoiesis.

## 5. Molecular Approaches Used for Exploration of Hematopoietic Niches

Certain molecular and cellular mechanisms are involved when it comes to the HSPSC niche. There has recently been a boom of technologies being used for the exploration of hematopoietic niches. The key features of the latest molecular approaches being implied in this research domain are summarized in Table 1. Single cell RNA-sequencing (scRNA-seq) is used widely to understand lineage differentiation in hematopoiesis. Certain methods used for scRNA-seq, including CEL-sq2, MARS-seq, Smart-seq, Smart-seq2, Drop-seq, and SCRB-seq, have been described to study large transcriptional differences in a few cells to understand the heterogeneity in cellular compartments [102,103]. Advances in scRNA-seq also enable the analysis of specific transcripts without restriction, and the heterogeneity among cell populations [104]. Furthermore, scRNA-seq of transgenic lines showed the involvement of ribosomal genes and lineage regulators in controlling hematopoietic differentiation, and revealed novel hematopoietic populations [105,106]. Moreover, a less conserved sequence of membrane proteins in NK cells as compared to T cells was observed while conducting comparative evolutionary studies on mammals and LCK-GFP transgenic zebrafish [107].

Zebrafish models have long been used for lineage tracing during embryonic stages, and recent genetic models have made that easier. For example, the lineage tracing of HSPCs from aortic hemogenic endothelium was performed using a multicolor transgenic labeling system called blood bow, along with high-end imaging and FACS. Labeling embryos with CRISPR/Cas9 and tracing hematopoietic clones revealed the generation of the hematopoietic system only from a group of cells present at the dome stage [108,109]. Recent transgenic approaches have also used the idea of recombinase-based techniques for genome editing using Cre/lox, ΦC31, and Flp/FRT in zebrafish to control transgene activity in order to study homeostasis and development, and to generate disease models [110].

Proteolytic environment in bone marrow is observed during HSC mobilization induced by cytokines or chemotherapy, and it influences the interaction of HSC with their niche chemotactically. This HSC mobilization causes an increase in protease, and a decrease in the endogenous level of protease inhibitors. Recently, zymographic analysis, active side labelling, and matrix-assisted laser desorption/ionization-time of flight (MALDI-TOF) analysis are techniques used for the detection of a proteolytic environment [111]. Further, certain stimulating agents tend to induce the transmigration of HSPC in the mobilization of HSPC or their interaction with the hematopoietic niche. Recently, the transwell migration assay has been developed to determine HSPC transmigration capacity and the interaction between hematopoietic cells and their niche [112]. Another method, different from microscopy-based screening, molecular level perturbation, and machine learning, was developed, which uses the concept of the dynamics of HSPC for testing novel mobilizing agents. The in vitro dynamic phenotyping method enables the classification of mobilization agents, and targets specific inhibitors/treatments quantitatively [113]. The hematopoietic microenvironment can also be constructed using morphological features using bone marrow granule samples and detailed images that illustrate hematopoietic cells with fibroblastic and histiocytic features [114].

The live imaging technique of slice cultures, clonal analysis, and mathematical modeling was used to understand the origin and 3-D organization of niches in intra-aortic hematopoietic cluster formation [115]. A combination of chemical screens with ScRNA-seq, in order to navigate therapy-resistant cells and to give an overview of the mechanisms exploring resistance to treatment in individual cells, may be the future cancer research avenue [25].

## 6. Computational Avenues for HSPCs Research Using CRISPR/cas9 Technology

Multiple tools are used for genome editing in zebrafish. A technology which has transformed reverse genetics in zebrafish is CRISPR/cas9 technology. It can be used to screen a large number of genes, and to generate disease models of study. Some of the salient features of the most-applied web-based tools are summarized in Table 2. The modern variant is Cas12a (Cpf1) [116]. CRISP-Cas9 technology uses a single guide RNA (sgRNA) for Cas9-mediated genome editing. One latest approach is SNP-CRISPR (https://www.flyrnai.org/tools/snp_crispr/ (2 February 2022)), which can be used to design sgRNA even with multiple SNPs [117]. Another web tool for genome editing is CRISPOR (http://crispor.org/ (2 February 2022). It uses guide RNAs as input sequence, and provides a whole solution for selection, cloning, the expression of guide RNA, and testing off-target mutations [118]. CRISPR-mediated gene editing can also be performed by CRISPR-ERA (http://CRISPR-ERA.stanford.edu (2 February 2022). It is a genome-wide sgRNA design tool for clustered regularly-interspaced short palindromic repeat mediated editing, repression, and activation [119]. Likewise, CRISPRdirect (http://crispr.dbcls.jp/ (2 February 2022) is used for designing CRISPR/Cas guide RNA with reduced off-target sites, and performs an exhaustive search against genome sequences [120]. After analyzing molecular features influencing sgRNA stability, activity, and loading in Cas9 in vivo, CRISPRscan (http://crisprscan.org/ (2 February 2022)was developed to design efficient sgRNAs [121]. Another web tool which is widely used in biomedical research to choose appropriate target sites is Cas-Designer (http://rgenome.net/cas-designer/ (2 February 2022); it provides all the possible guide RNA sequences in the given DNA sequence and their off-targets [122]. CRISPR guide RNA libraries and the editing of coding and non-coding genomic regions of RNA can be performed by GuideScan software (http://guidescan.com/ (2 February 2022); it provides more specific gRNA design [123]. Another tool for sgRNA design is AlleleAnalyzer (https://github.com/keoughkath/AlleleAnalyzer (2 February 2022), which incorporates single nucleotide variants and short deletions and insertions for sgRNA designing for precise editing of one or multiple haplotypes of a sequenced genome [124]. The accurate precision of sgRNA on target knockout efficacy and off-target profile is a major challenge in CRISPR systems. To overcome this, DeepCRISPR (http://www.deepcrispr.net/ (2 February 2022) was developed to identify sequence and epigenetic features which can affect sgRNA knockout efficacy [125].

To understand the fate specification of neural crest stem cells and functions of Tfec, genome editing was done using version 1 of the webtool, CHOPCHOP (http://chopchop.cbu.uib.no/ (2 February 2022). The CHOPCHOP webtool identifies CRISPR-Cas sgRNA targets, and has expanded its toolbox beyond knockouts with a recent version, CHOCHOP v3 (129). To this end, advanced innovation relying on currently available methods will exaggerate precision, and accuracy should be pursued to combat these challenges.

## 7. Concluding Remarks and Future Prospectus

Recent advances of experimental technologies, including gene editing tools and sequencing technologies, have empowered researchers to explore the role of genetic and epigenetic factors responsible for hematopoietic maintenance and its regulation. HSCs are conserved in nature, but the role of different signaling events at different hematopoietic niches play a pivotal role for hematopoietic maintenance and quiescence by up-regulating or suppressing specific genes. Even though there are intensive studies that have been conducted to delineate the hematopoietic niches, there is still controversy about the location of HSCs, and the role of other niche cells and how they communicate with each other through systemic signaling mechanisms. RNA-binding proteins are well characterized for their role in HSCs initiation, but how different RNA-binding proteins are linked together to exert an effect for the regulation of niche cells still needs to be addressed. There are recently available molecular approaches and web-based tools that enable gene editing technology to work efficiently, but future investigations for improved precision and sensitivity should be pursued to combat the current challenges. Furthermore, in the future, HSC can also be used in the reconstitution of damaged bone marrow (BM), and, as a result, would be helpful for the treatment of non-blood diseases.

## Figures and Tables

**Table 1:** Recent molecular approaches employed for functional characterization.

Molecular Technique	Functional Exploration	References
Single cell RNA-sequencing CEL-sq2 MARS-seq Smart-seq Smart-seq2 Drop-seq SCRB-seq	Lineage differentiation in hematopoiesis Analysis of specific transcripts without restriction and heterogeneity among cell populations Involvement of ribosomal genes and lineage regulators in controlling hematopoietic differentiation	[102,103] [104] [105,106]
Blood bow High end imaging FACS CRISPR/Cas 9	Lineage tracing of HSPCs of aortic endothelium	[108,109]
Recombinase based techniques for genome editing using Cre/lox, ΦC31, and Flp/FRT	To control transgene activity to study homeostasis and development and disease models	[110]
Zymographic analysis, active side labelling and MALDI-TOF	To observe proteolytic environment during HSC mobilization	[111]
Transwell migration assay	Determines HSPC transmigration capacity and interaction between hematopoietic cells and their niche	[112]
In vitro dynamic phenotyping method	Quantitative classification of mobilization agents and target specific inhibitors/treatments using dynamics of HSPC	[113]
Morphological features	Construction of hematopoietic microenvironment	[114]
Live imaging technique of slice cultures, clonal analysis, and mathematical modeling	Understanding origin and 3-D organization of niche in intra-aortic hematopoietic cluster formation	[115]

**Table 2:** Web-based tools used for HSPCs research avenues.

Web tool	Website	Features	Reference
SNP-CRISPR	https://www.flyrnai.org/tools/snp_crispr/ (2 February 2022	Allows to design sgRNA with multiple SNPS	[117]
CRISPOR	http://crispor.org/ (2 February 2022)	Comprehensive solution for selection, cloning, expression of guide RNA, and testing off-target mutations	[118]
CRISPR-ERA	http://CRISPR-ERA.stanford.edu (2 February 2022)	sgRNA design tool for clustered regularly-interspaced short palindromic repeat mediated editing, repression, and activation	[119]
CRISPRdirect	http://crispr.dbcls.jp/ (2 February 2022)	Designing CRISPR/Cas guide RNA with reduced off-target sites	[120]
CRISPRscan	http://crisprscan.org/ (2 February 2022)	In vivo efficient sgRNAs design	[121]
Cas-Designer	http://rgenome.net/cas-designer/ (2 February 2022)	To choose appropriate target sites	[122]
GuideScan	http://guidescan.com/ (2 February 2022)	More specific gRNAs design	[123]
AlleleAnalyzer	https://github.com/keoughkath/AlleleAnalyzer (2 February 2022)	Precise editing of one or multiple haplotypes of a sequenced genome	[124]
DeepCRISPR was developed	http://www.deepcrispr.net/ (2 February 2022)	Identifies sequence and epigenetic features which can affect sgRNA knockout efficacy	[125]
CHOPCHOP v3	http://chopchop.cbu.uib.no/ (2 February 2022)	Identifies CRISPR-cas sgRNA targets	[126]

## Data Availability

Not applicable.
